# Using network analysis to identify leverage points based on causal loop diagrams leads to false inference

**DOI:** 10.1038/s41598-023-46531-z

**Published:** 2023-11-29

**Authors:** Loes Crielaard, Rick Quax, Alexia D. M. Sawyer, Vítor V. Vasconcelos, Mary Nicolaou, Karien Stronks, Peter M. A. Sloot

**Affiliations:** 1 Department of Public and Occupational Health, Amsterdam UMC Location University of Amsterdam, Amsterdam, The Netherlands; 2 Institute for Advanced Study, University of Amsterdam, Amsterdam, The Netherlands; 3 Computational Science Lab, Informatics Institute, University of Amsterdam, Amsterdam, The Netherlands; 4 MRC Epidemiology Unit, Institute of Metabolic Science, University of Cambridge School of Clinical Medicine, Cambridge, UK; 5 POLDER, Institute for Advanced Study, University of Amsterdam, Amsterdam, The Netherlands; 6 Center for Urban Mental Health, University of Amsterdam, Amsterdam, The Netherlands

**Keywords:** Public health, Complex networks, Computational science

## Abstract

Network analysis is gaining momentum as an accepted practice to identify which factors in causal loop diagrams (CLDs)—mental models that graphically represent causal relationships between a system’s factors—are most likely to shift system-level behaviour, known as leverage points. This application of network analysis, employed to quantitatively identify leverage points without having to use computational modelling approaches that translate CLDs into sets of mathematical equations, has however not been duly reflected upon. We evaluate whether using commonly applied network analysis metrics to identify leverage points is justified, focusing on betweenness- and closeness centrality. First, we assess whether the metrics identify the same leverage points based on CLDs that represent the same system but differ in inferred causal structure—finding that they provide unreliable results. Second, we consider conflicts between assumptions underlying the metrics and CLDs. We recognise six conflicts suggesting that the metrics are not equipped to take key information captured in CLDs into account. In conclusion, using betweenness- and closeness centrality to identify leverage points based on CLDs is at best premature and at worst incorrect—possibly causing erroneous identification of leverage points. This is problematic as, in current practice, the results can inform policy recommendations. Other quantitative or qualitative approaches that better correspond with the system dynamics perspective must be explored.

## Introduction

Over the past two decades, the study of complex health problems from a systems dynamics perspective has become increasingly widespread. The importance of shifting towards systems thinking to understand how to address complex problems has been recognised in mental health^1^, public health^2^, and planetary health^3^. Complex problems are characterised by the involvement of many diverse factors from many different domains that relate to each other in ways that are difficult to predict^4^. Taking a systems dynamics perspective means looking at these factors and their interactions as constituting a system that operates across domains, from cells to society^5^. The causal loop diagram (CLD) is increasingly valued as an approach to graphically represent how factors in a system causally relate to each other. It serves as the first step towards understanding the system behind a complex problem (see health-related examples^6–14^, illustrative examples will also be presented and interrogated in this paper) and, accordingly, how to address that problem—typically with a complex intervention^15,16^.

The CLD is a “tool to map the feedback structure of complex systems”^17^—where a feedback loop refers to the output of a factor also serving as an input to that factor^18^—and can be formulated based on specialised knowledge, experiential knowledge, and/or literature. The CLD stems from system dynamics practice^17,19^ and is “most fundamentally (…) a way of surfacing, visualising, and exploring mental models”^19^. Sterman, a leading researcher in system dynamics, describes, in one of the seminal texts of the field, that the term ‘mental model’ covers “our beliefs about the networks of causes and effects that describe how a system operates, along with the boundary of the model (which variables are included and which are excluded) and the time horizon we consider relevant—our framing or articulation of a problem”^17^. The CLD’s intended uses are “quickly capturing your hypotheses about the causes of dynamics”, “eliciting and capturing the mental models of individuals or teams”, and “communicating the important feedbacks you believe are responsible for a problem”^17^.

To achieve this, in a CLD, causal effects of a factor X on a factor Y are depicted as arrows, where the direction of the arrow indicates which factor is the cause, X, and which factor experiences the effect, Y. The arrows are accompanied by polarities to represent positive—if X increases, Y increases—or negative—if X increases, Y decreases—effects, respectively indicated with ‘+’ and ‘−’^20^. Since a CLD is best described as a graphical representation of a mental model developed for a specific problem setting, as outlined above, it follows that the inferred causal structure of a CLD—consisting of factors, arrows representing causal effects, and polarities showing whether causal effects are positive or negative—is dependent on those involved in its formulation and the spatial and temporal scales of interest^7,20,21^. This sets the CLD apart from causal models that primarily focus on delineating the aetiology of a problem, where the CLD—albeit in many cases substantiated with (scientific) evidence—first and foremost captures what individuals or teams perceive to be the underpinnings of a problem.

The final objective of developing a CLD is typically to identify places to intervene in the system where “a small change could lead to a large shift in [system-level] behaviour”^22^, referred to as leverage points. Currently in the literature relating to the development and analysis of CLDs, the term ‘leverage point’ tends to be used as referring to a factor within the CLD “in which change is likely to yield significant shifts elsewhere in the system”^23^. The key premise is that the system depicted in the CLD is producing undesirable system-level behaviour, and that there are central drivers in the system that could be influenced in such a way that this undesirable system-level behaviour is sustainably disrupted^8,24–30^.

Thus far, the identification of leverage points on the basis of CLDs has mostly relied on qualitative interpretation, where based on visual inspection of the causal structure of the CLD researchers can hypothesise about system-level behaviour and which factors are most important in explaining it^18,31,32^. Many researchers however recognise the advantages of quantitatively identifying leverage points based on CLDs: because of the numerous, often non-linear, interactions between the factors involved, computers are better equipped than people to systematically observe the entire causal structure^22,33^.

Still, quantitatively identifying leverage points based on CLDs has proven difficult. System dynamics modelling and other computational modelling approaches, which translate the CLD into a set of mathematical equations^34^, can be used to simulate the consequences of intervening on a (set of) factor(s) for system-level behaviour and thus to identify leverage points^22^—but they come with significant challenges. These stem from the specialist computational modelling expertise required to bridge experience, theorisation, and its mathematical application. This expertise requires substantial resources, is hard to come by, and may not be available at all in some contexts in which systems thinking is traditionally applied, such as community settings^20^. In addition, there is an imbalance between the quantitative data available on individual versus environmental factors, with the latter being underrepresented^2^.

In seeking alternative methods, network analysis^35^ has gained momentum as an accepted practice to quantitatively identify leverage points based on CLDs^8,23–30,36–55^—for example in public health^24,38,45,50,52,53,55^. Here, 22 of the 29 papers that we identified that adopt this practice were published in 2021 (4 papers^23,25,37,50^), 2022 (8 papers^27,28,43–45,47,49,52^), and 2023 (10 papers to date^26,30,36,38,39,41,42,46,53,55^). Network analysis seemingly has the potential to mathematically analyse a CLD, which in this context can be considered a network of factors, to systematically find which factors represent leverage points. It is an attractive alternative to computational modelling approaches to quantitatively identify leverage points because it can be conducted based solely on the CLD, can be performed using easy-to-use software tools—requiring neither quantitative data nor computational modelling expertise—and always produces a result, i.e., a ranking of the CLD’s factors from least to most important for intervention. Specifically, network analysis is based on graph theory, a mathematical discipline that aims to study the properties of networks^56^. Network analysis allows for the quantification of a network’s global properties, e.g., the overall density of the network, and its local properties, e.g., the importance of single factors within the network^56^. It is conventionally applied to social networks, where each node represents a person (compare factor in the CLD) and each edge represents a social relationship (compare causal effect in the CLD)^57^ and has been extended to e.g., computer networks and biological networks^58^.

The overarching approach taken in using network analysis to identify leverage points based on CLDs is to translate the CLD’s factors and arrows into an adjacency matrix, after which network analysis metrics can be employed. Metrics commonly applied in combination with CLDs are betweenness centrality^8,23–27,29,38–45,48,50–55^ and closeness centrality^8,23,26,29,39,42,43,50,52,53^. The factor with the highest betweenness centrality lies on the highest number of shortest causal chains between pairs of other factors in the CLD^58^, hypothesised to indicate that it is an important mediator^8,24,26,29,38,42^. The factor with the highest closeness centrality on average has the shortest causal chains to other factors^58^, reasoned to signal its importance as a spreader of causal power^8,26,29,42^. In the context of CLDs, the rationale behind using these metrics, which rely on a factor’s position on short causal chains, is that one could assume that causal power gradually diminishes while going down a causal chain^8^, making interventions on the factors that are involved in short causal chains the most likely to shift system-level behaviour. That is, the hypothesis is that the shorter the causal chain between a factor X and a factor Z, the more of the causal power from a change in X is left when we get to Z—and, thus, the more causal power is exercised on Z by X.

In current practice, the results obtained from applying betweenness- and closeness centrality, among other network analysis metrics, to CLDs have been utilised to inform policy recommendations. Examples of this are given in Table 1^8,24,42,45,50,52,55^. In this context, factors with the highest rankings on betweenness- and/or closeness centrality, among other network analysis metrics, are presented as places in the system for which effective intervention is likely to have the largest impact on specified system outcomes.

**Table 1:** Examples of how the results obtained from applying betweenness- and closeness centrality, among other network analysis metrics, to CLDs are utilised to inform policy recommendations in current practice.

Citation	How the results obtained from applying network analysis to CLDs are utilised to inform policy recommendations
Hoyer et al.^42^	“through the application of network analysis, we determined the specific properties of the causal loop diagram to derive potential intervention points in the network to introduce and spread change more efficiently”
Koorts et al.^45^	“optimising those types of variables that have interconnectedness with others in a system, and targeting them as ‘leverage points’ (…), may be one way of effectively changing system outcomes to achieve more sustainable impacts on broader population health”; “the rationale being that it enables examination of not only the direct effect of an intervention or exposure on active recreation, but also identifies the indirect effects on active recreation via wider system features”
McGlashan et al.^24^	“for population health problems, the insight of central variables can aid intervention planning by understanding their role in the system”; “insight from network analysis can aid community groups in intervention design by considering a variable’s position in the network”
Savi et al.^50^	“the calculation of the metrics combined with the properties of the network enables the identification of potential strategies that may guide policy recommendations for better control of malaria”
Smith et al.^52^	“leverage points can be identified through topological analysis of the system map structure”; “these leverage points become the focus of interventions that could promote equitable use of urban blue spaces”
Uleman et al.^8^	“high centrality for modifiable risk factors such as social relationships and physical activity (…) suggest that they may be promising leverage points for interventions”
Zucca et al.^55^	“rather than trying to intervene across the whole system of nature-based early learning and childcare centres delivery it may be most rewarding to invest available resources in subsections of the system, focusing on the small number of leverage points identified in this study as being the most important/applicable to the context of the any given early learning and childcare centres practice”

While its supposed advantages have led to a growing trend in the use of network analysis to identify leverage points based on CLDs, this application of network analysis has to our knowledge not been duly reflected upon. Yet, evaluations regarding the utility of network analysis if applied to a range of other types of networks and (causal) models have shown that the assumption that betweenness- and closeness centrality are equally indicative of a factor’s importance in for example psychological networks, directed acyclic graphs, and dynamical systems as they are in social networks is far from trivial^59–61^. Evaluating the utility of network analysis in the identification of leverage points based on CLDs is critical as the results provided by this method can, in current practice, form the foundation for policy recommendations about how to address complex problems.

In this paper, we evaluate whether it is justified to use betweenness- and closeness centrality to identify leverage points based on CLDs. To this end, first, we assess whether betweenness- and closeness centrality identify the same leverage points based on CLDs that represent the same system but differ in inferred causal structure. In other words, we assess whether the metrics provide reliable results. Second, we consider conflicts between the assumptions underlying betweenness- and closeness centrality and CLDs to understand whether the current practice of applying these metrics to CLDs is theoretically sound.

## Results

### Betweenness- and closeness centrality do not provide reliable results

**Figure 1:**
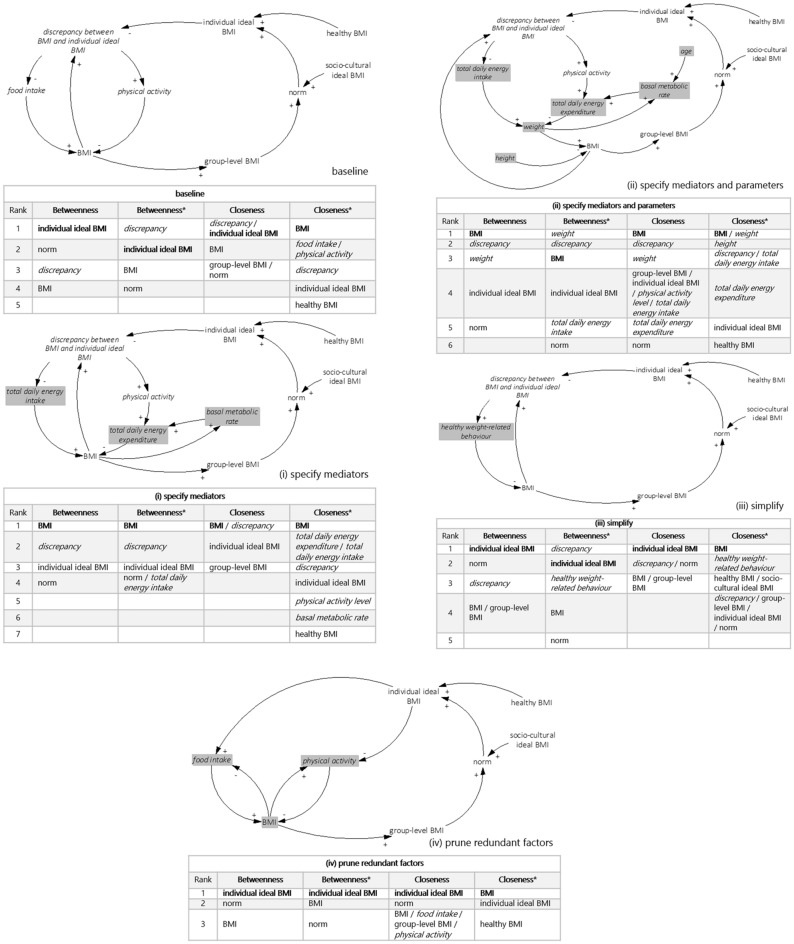
The baseline CLD and the four alternative versions of the baseline CLD with their highest ranking factors on betweenness- and closeness centrality (the undirected and directed (indicated with *) variants). The adjustments as compared to the baseline CLD are highlighted in each of the four alternative versions. The highest ranking factors are reported until they include the three highest ranking factors from the subset of factors that are included in all five CLDs. The highest ranking factor from this subset is indicated in bold. Factors that are not included in all five CLDs are indicated in *italics*.

To assess whether betweenness- and closeness centrality provide reliable results, we compute the metrics for five CLDs (Fig. 1)—a baseline CLD and four alternative versions of that baseline CLD—that differ in causal structure but represent the same system. The baseline CLD is a simplified variant of a previously published CLD^62^. Considering that a CLD is a graphical representation of a mental model developed for a specific problem setting, the five CLDs differ in causal structure due to modelling choices that can be made one way or another depending on the research question as well as the modeller(s)^20,21^, with each choice being justifiable. Table 2 details the modelling choices made in each of the four alternative versions of the baseline CLD: (i) specify mediators, (ii) specify mediators and parameters, (iii) simplify, and (iv) prune redundant factors.

**Table 2:** The four alternative versions of the baseline CLD with corresponding modelling choices and adjustments to the baseline CLD.

Alternative version	Adjustments to the baseline causal loop diagram
(i) Specify mediators	**Modelling choice: make the underlying mechanisms in the causal chains between *discrepancy between BMI and individual ideal BMI* and *BMI* explicit by adding mediators to the baseline CLD (also carried out in this paper**^62^**)** Adjustments: Replace *food intake* and *physical activity* by *total daily energy intake* and *total daily energy expenditure*, via *physical activity*, respectively, to clarify that changing weight-related behaviour impacts BMI via a change in energy balance^63^: reducing energy intake and increasing energy expenditure, which can both be measured and computed in kilocalories Include causal effects between *BMI* and *basal metabolic rate* and between *basal metabolic rate* and *total daily energy expenditure*: more energy is required to sustain body function at rest when BMI is higher (note that this mechanism is added because you only notice that this mechanism should be accounted for once you include *total daily energy expenditure* as a mediator)^64^
(ii) Specify mediators and parameters	**Modelling choice: make the underlying mechanisms in the causal chains between *discrepancy between BMI and individual ideal BMI* and *BMI* explicit by adding mediators to the baseline CLD and add the parameters that would be necessary to compute the included factors (also carried out in this paper**^62^**)** Adjustments: Replace *food intake* and *physical activity* by *total daily energy intake* and *total daily energy expenditure*, via *physical activity*, respectively, to clarify that changing weight-related behaviour impacts BMI via a change in energy balance^63^: reducing energy intake and increasing energy expenditure, which can both be measured and computed in kilocalories Include causal effects between *BMI* and *basal metabolic rate* and between *basal metabolic rate* and *total daily energy expenditure*: more energy is required to sustain body function at rest when BMI is higher (note that this mechanism is added because you only notice that this mechanism should be accounted for once you include *total daily energy expenditure* as a mediator)^64^ Include the parameters *age* and *height* as factors in the causal structure as, besides weight, they are required to compute *basal metabolic rate* and *BMI*, respectively Alter the causal structure to incorporate *weight* in addition to *BMI* because *basal metabolic rate* is numerically estimated based on weight in kg and not BMI in kg/m^2^
(iii) Simplify	**Modelling choice: simplify the baseline CLD** Adjustments: Merge the two feedback loops that are present in the baseline CLD, between *norm* and *food intake* and between *norm* and *physical activity*, into one feedback loop between *norm* and *healthy weight-related behaviour*
(iv) Prune redundant factors	**Modelling choice: prune factors from the baseline CLD that are not required for the computation of the included factors** Adjustments: Remove the factor *discrepancy between BMI and individual ideal BMI*, because this factor is merely a redefinition based on two other factors, *BMI* and *individual ideal BMI*, that are already present in the baseline CLD and is therefore computationally not required to be formulated as a separate factor (i.e., it is an auxiliary variable^21^) Alter the causal structure so that *food intake* and *physical activity* are influenced by *BMI* and *individual ideal BMI* directly, as opposed to via *discrepancy between BMI and individual ideal BMI*, which is numerically equivalent to the baseline CLD, as shown below: if we were to build two system dynamics models with mathematical equations, one based on the baseline CLD and one based on this alternative version, the system dynamics models would be identical—and, thus, generate identical simulation results exemplifying system-level behaviour baseline* $discrepancy \; between \;BMI \; and \;individual \; ideal \;BMI=BMI-individual \; ideal \;BMI$ $food\; intake={c}_{1}\times discrepancy \;between \;BMI \; and\; individual \; ideal \; BMI$ $physical \; activity={c}_{2}\times discrepancy \; between \;BMI \;and \; individual \; ideal \; BMI$ (iv) prune redundant factors* $food \; intake={c}_{1}\times (BMI-individual \; ideal \; BMI)$ $physical \; activity={c}_{2}\times (BMI-individual \; ideal \; BMI)$ *c_*1*_ and *c*_*2*_ are parameters converting *BMI* into *food intake* and *physical activity* units, respectively

Figure 1 shows the highest ranking factors on betweenness- and closeness centrality for the baseline CLD and the four alternative versions (the lower ranking factors can be found in Supplementary Table S1 online). Because only the factors *BMI*, *group-level BMI*, *healthy BMI*, *individual ideal BMI*, *norm*, and *socio-cultural ideal BMI* are included in all five CLDs, we report the highest ranking factors until they include the three highest ranking factors from this subset of factors—so that the differences between the CLDs can be compared directly. Still, the full rankings are useful to consider as well, as in reality one could argue that all factors would be included in the ranking—because it would be unknown how alternative versions of the CLD could look and thus which factors would and would not be consistent across all alternative versions. Note that the metrics are normalised to account for differences in the number of factors included in the CLDs. The undirected as well as directed variants (the directed variants are indicated with *) of betweenness- and closeness centrality are assessed, since both are being used to identify leverage points based on CLDs—as is further explained in the next section. Applying the undirected variants means assuming that causal effects act in both directions, disregarding the directions of the arrows, while applying the directed variants means assuming that causal effects act in one direction, as per the directions of the arrows.

The adjustments made to the baseline CLD to meet the various modelling choices as described in Table 2 shift the rankings for betweenness centrality, betweenness centrality*, and closeness centrality, even though the CLDs represent the same system. Notably, the ranking for closeness centrality*—when looking at the highest ranking factor and disregarding the factors that are not included in all five CLDs—is consistent over the CLDs, except for alternative version (iii).

In addition to the rankings not being consistent over the CLDs, the results also highlight that the ranking of the factors for a single CLD is dependent on whether the undirected or directed variants of the metrics are used. For betweenness centrality, the undirected and directed variants agree on only two of the five rankings; for closeness centrality, they agree on none of the rankings.

The ranking of the factors from least to most important as determined according to betweenness centrality, betweenness centrality*, and closeness centrality is thus inconsistent over the CLDs, while closeness centrality* is mostly consistent. Betweenness- and closeness centrality, with the exception of closeness centrality*, do not provide reliable results: they identify different leverage points based on CLDs that represent the same system.

### Betweenness- and closeness centrality are not equipped to take key information captured in causal loop diagrams into account

In order to understand whether the current practice of applying the metrics to CLDs is theoretically sound, we consider conflicts between the assumptions underlying betweenness- and closeness centrality and CLDs. The conflicts we recognise are sixfold, as listed below, and further discussed in the next sections. The fundamental issue underlying each of the six conflicts is that betweenness- and closeness centrality are not equipped to take key information that is captured in CLDs into account.

1. Undirected variants of betweenness- and closeness centrality are being used, while *CLDs rely on arrows to describe the directions of causal effects*
2. Betweenness- and closeness centrality do not take polarities into account, while *CLDs rely on polarities to describe whether a causal effect is positive or negative*
3. Applying betweenness- and closeness centrality means assuming that what flows through the network takes the shortest path, while *what flows through CLDs may not take the shortest path*
4. Applying betweenness- and closeness centrality means assuming that all factors in the CLD belong to the same domain, while *CLDs include factors from many different domains*
5. Applying betweenness- and closeness centrality means assuming that there is no overlap between the factors in the CLD, while *CLDs are used to show interactions between lower and higher domains*
6. Betweenness- and closeness centrality cannot tell us how interventions on different factors interact, while *CLDs are developed to inform complex interventions with interacting components*

#### Causal loop diagrams rely on arrows to describe the directions of causal effects

**Figure 2:**
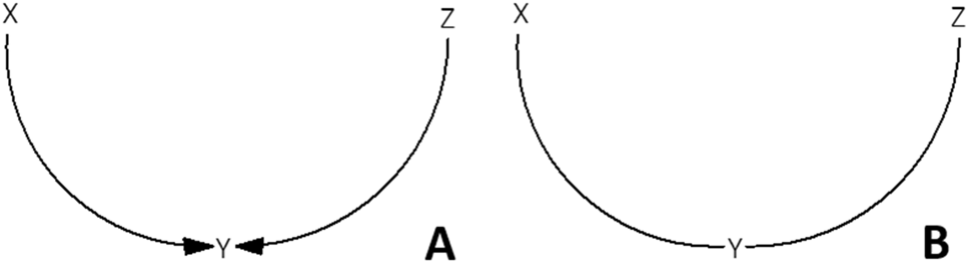
Using undirected variants of betweenness- and closeness centrality means loss of the information represented by the directions of the arrows.

Undirected variants of betweenness- and closeness centrality are occasionally being used to identify leverage points based on CLDs, while applying these variants means assuming that edges are undirected. When these variants are used, each edge is interpreted as a line rather than as an arrow: causal effects act in both directions rather than in one direction^58^. CLDs, however, characteristically have directed edges, i.e., arrows, which represent the causal effect of one factor on another factor^20^. Using undirected variants of betweenness- and closeness centrality means loss of the information represented by the directions of the arrows. For example (Fig. 2), a CLD may indicate that a factor X and a factor Z each assert a causal effect on a factor Y—using two directed edges (Panel A). Using the undirected versions of the metrics, these two edges would connect X to Z (Panel B). Reasoning from the arrows, however, Z could not be reached from X. In the interest of the identification of leverage points, using the undirected variants of betweenness- and closeness centrality could erroneously lead to the conclusion that intervening on X could result in a change in Z.

#### Causal loop diagrams rely on polarities to describe whether a causal effect is positive or negative

Because they do not take polarities^58^ into account, betweenness- and closeness centrality do not tell us in which direction, increase/positive or decrease/negative, to intervene on a leverage point in order to engender change. While the metrics are reasoned to indicate whether a factor is a leverage point, it may not actually be possible to intervene on an identified leverage point such that it shifts system-level behaviour in a way that is intended.

**Figure 3:**
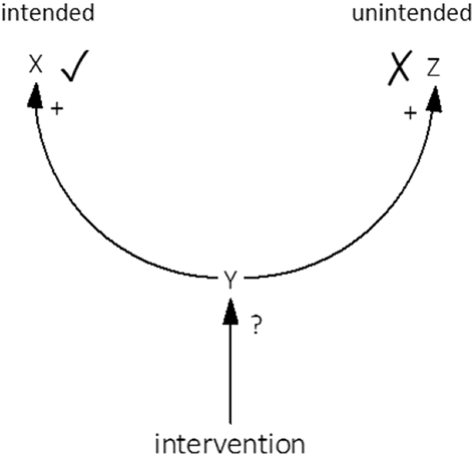
It may not actually be possible to intervene on an identified leverage point such that it shifts system-level behaviour in a way that is intended.

To make this concrete, consider three factors in a system: X, Y, and Z (Fig. 3). Suppose that Y is identified as a leverage point because it has a causal effect on all other factors, i.e., X and Z. Even though Y has a causal effect on both X and Z, the causal effects are not necessarily both in the direction that we intend. X as well as Z are positively influenced by Y, i.e., an increase in Y causes an increase in X as well as an increase in Z, but maybe what we are looking to achieve is only an increase in X and not in Z. It is then impossible to develop an intervention on Y that influences both X and Z in the intended direction. That is, if we increase or decrease Y, we influence either X or Z in the opposite direction of what we aimed for: there is one intended and one unintended consequence. This means that intervening on a factor that is identified as a leverage point can result in a net intervention effect of zero, which implies that it does not qualify as a leverage point as it does not shift system-level behaviour.

#### What flows through causal loop diagrams may not take the shortest path

Applying betweenness- and closeness centrality relies on the assumption that what is flowing through the network, first, knows the shortest path through the network to reach its destination and, second, takes the shortest path to reach that destination^65^. When what flows through the network does not abide by these assumptions, the metrics may identify a node as important while it is actually not important for the flow process under study^65^. Specifically, the importance of a node depends on both the structure of the network and the flow process that the edges represent^65^. Even though all networks can be demonstrated as a structure of nodes and edges, a node that is “structurally important” in one network with one type of flow process is not necessarily “structurally important” in another network with another type of flow process^59,60,66^. For example, betweenness- and closeness centrality have been shown to correctly identify important nodes if the flow process under study is the delivery of a package, where the person delivering the package knows where they need to go and the most efficient way to get there. However, that is not the case if the flow process under study is an infectious disease, where the assumptions of knowing and taking the shortest path are unbefitting^65^.

As CLDs are graphical representations of mental models of the important feedbacks believed to be responsible for a problem^17^, what flows through a CLD is best described as ‘causality’ or ‘causal impact’, where a change in a factor propagates through all allowed paths with different intensities and, at a given timescale, may even reinforce or suppress itself. Hence, it may be at best premature and at worst incorrect to use metrics that imply the assumption that what flows through a CLD relies on knowing and taking the shortest path. However, this is the implicit assumption being made when applying betweenness- and closeness centrality to CLDs. As a result, the factors that are identified as mediators by betweenness centrality and as spreaders of causal power by closeness centrality may not actually have these roles in the type of flow process that is represented in CLDs and may therefore not qualify as leverage points.

#### Causal loop diagrams include factors from many different domains

Applying betweenness- and closeness centrality means assuming node exchangeability, which is the premise that the only reason that a node may be more important than another node for system-level behaviour stems from its different position in the network, i.e., there are no other differences in node characteristics^61^. Contrary to social networks, where each node corresponds to a person, in CLDs the nodes *do* have different characteristics: they represent many diverse factors from many different domains and with various units of measurement. When factors from many different domains are included in the network it becomes less likely that the assumption of node exchangeability holds. This is because the factors in higher domains may, for example, have a larger or faster causal effect on the factors in lower domains—e.g., society on cells or the environment on an individual—than vice versa—e.g., cells on society or an individual on the environment—while they are connected in the same way^67^. In CLDs, the importance of a node for system-level behaviour thus does not exclusively stem from its position in the network but also from the domain in which it operates. By making the assumption of node exchangeability, as we do when we use betweenness- and closeness centrality, factors that presumably are less likely to shift system-level behaviour, such as those in lower domains, are regarded as equivalent to factors that presumably are more likely to shift system-level behaviour, such as those in higher domains.

#### Causal loop diagrams are used to show interactions between lower and higher domains

Applying betweenness- and closeness centrality means assuming that there is no conceptual overlap between the factors in the CLD, an assumption that is generally required for network analysis^61,68^. To use the metrics there should be node distinctiveness, where nodes are causally related and independent rather than constitutively related and overlapping^68^. In social networks this assumption tends to be satisfied because people are distinct entities^61^. In CLDs, on the other hand, it may be difficult to ensure that nodes do not overlap. Notably, it has been argued that guaranteeing node distinctiveness becomes increasingly more difficult as networks include factors that operate across a greater variety of domains^68^. Yet, the aim of a CLD is exactly this: to graphically represent how factors in a system that operates across domains causally relate to each other. For example, a CLD could include factors pertaining to the local food environment—e.g., special offers on unhealthy foods in a local supermarket—as well as factors pertaining to the global food environment—e.g., global marketing strategies—in the same causal structure, where the local food environment could be considered part of the global food environment. In other words, the global food environment contains the local food environment, but factors pertaining to both may be included separately in the network, which is difficult to prevent if many different domains are covered. If we do assume node distinctiveness even if there is conceptual overlap between the factors in the CLD so that we can use betweenness- and closeness centrality, we may identify leverage points that are not independent of other factors in the CLD^68^. Accordingly, we may then not be able to intervene exclusively on the identified factor^68^. For example, suppose we identify global marketing strategies as the factor to intervene on, but we have developed the CLD based on the premise that special offers on unhealthy foods in a local supermarket are also a component of the global food environment. Then it is unclear how we should separate the global food environment from what is happening in local supermarkets and thus what it means that one but not the other is identified as a leverage point.

#### Causal loop diagrams are developed to inform complex interventions with interacting components

If betweenness- and closeness centrality are used to identify leverage points based on CLDs, then it is implicitly assumed that leverage points can be computed in isolation. That is, the metrics do not provide us with any information about how interventions on multiple factors in the CLD may interact—for example about whether two interventions might function in synergy, where the effect of the interventions combined is greater than the sum of the effects of each intervention separately. This issue is not resolved by simultaneously intervening upon several factors identified as leverage points by the metrics, such as the three highest ranking factors, as applying the metrics means assuming that interventions occur in only one leverage point at a time. For example, if a factor X and a factor Y both score high on closeness centrality, the results only tell us that intervening on X *or* Y is relevant, while an intervention on both X *and* Y has an unknown result as we do not know how the interventions interact with each other—they may even cancel each other out. Betweenness- and closeness centrality thus cannot advise us on coordinated interventions that address multiple factors in the CLD at the same time, while CLDs are typically used as a starting point for the development of complex interventions^15^, which are “interventions that contain several interacting components”^16^.

## Discussion

The aim of this paper was to evaluate whether it is justified to use betweenness- and closeness centrality to identify leverage points based on CLDs by assessing whether the metrics provide reliable results and considering conflicts between the assumptions underlying the metrics and CLDs. We found that, in current practice, this application of network analysis is neither reliable nor theoretically sound. First, betweenness- and closeness centrality, with the exception of the directed variant of closeness centrality, identify different leverage points based on CLDs that represent the same system. While the directed variant of closeness centrality in this case unexpectedly showed consistency over the different versions of the CLD, the theoretical conflicts uncovered suggest that this might be coincidental. For another set of alternative versions of this CLD it is possible that one of the other metrics would be more reliable. The key finding is that network analysis metrics are highly sensitive to changes made to a CLD. This means that, when network analysis is used, different leverage points may be identified due to modelling choices that can be made one way or another, depending on the research question as well as the modeller(s), and that do not alter the system that is represented by the causal structure. It is however natural for such differences between CLDs to occur, since CLDs are graphical representations of mental models developed for specific problem settings^7,20,21^. Second, we recognise six conflicts between the assumptions underlying betweenness- and closeness centrality and CLDs, where, as we have described, each of these conflicts can result in erroneous identification of a factor as a leverage point. Even if betweenness- and closeness centrality were to provide reliable results, the six conflicts recognised give us reason to believe that the metrics may still leave us with ‘the wrong answer’ in terms of leverage points. Specifically, the fundamental issue underlying each of the six conflicts is that betweenness- and closeness centrality are not equipped to take key information that is captured in CLDs into account.

Wrongly identifying a factor as a leverage point can have significant implications, as the results provided by betweenness- and closeness centrality can, in current practice, form the foundation for policy recommendations about how to address complex problems. Consider for example the results for betweenness centrality for the baseline CLD and alternative version (i) as an illustration of the consequences of shifts in the ranking for policy recommendations. By adding mediators to the baseline CLD, which is the modelling choice made in alternative version (i), individual-level BMI becomes a more important leverage point than the group-level norm, while BMI appears less important than the norm based on the causal structure of the baseline CLD. If we consider that the factors identified refer to potential places to intervene in the system, targeting the group-level norm, for example by community-wide campaigns promoting a healthy diet, warrants a fundamentally different course of action in terms of policy than targeting individual-level BMI, which could, for example, imply supporting people to individually alter their diet.

The problems with the use of betweenness- and closeness centrality to identify “structurally important nodes”^66^ are far from unfamiliar, where many of the problems previously detected in other fields^61^ also apply to CLDs and several papers applying network analysis metrics to CLDs also hint at potential limitations^8,23,24,26,28,36–38,42,52^. It has already been shown for different types of networks that betweenness- and closeness centrality do not provide reliable results over networks that differ slightly in structure, for example when a node is included or excluded^61,69,70^. As for the conflicts between assumptions, the fact that betweenness- and closeness centrality rely on knowing and taking the shortest path has been criticised even for social networks, where a person due to social preferences may refrain from sharing information with some people in the network and not others, causing information to take a longer path than theoretically necessary^61,65,66^. Tellingly, investigating the conceptual underpinnings of psychological networks and the associated methods is a discipline in itself in the field of psychology^61,71^. For social networks and psychological networks, scrutiny of these metrics has even gone a step further by testing whether real-world interventions on the nodes that the metrics identified as important indeed had a large effect on system-level behaviour, which also did not lead to the anticipated results^61^. For social networks, it has for example been shown that removing an identified structurally important person did not weaken but rather strengthened the network^72^, while for psychological networks there are indications that an identified structurally important symptom does not necessarily have high predictive power for system-level behaviour^73^. Challenges regarding the specification of boundaries—i.e., which nodes should be included in and excluded from the network—which critically affects betweenness- and closeness centrality results, have accordingly also been extensively discussed in other fields^69,74,75^. This and other bodies of literature do not seem to have been considered in research using CLDs, presumably because, even though network analysis metrics are being applied, CLDs are not typically thought of as analysable as networks, with many papers that apply network analysis metrics to CLDs first providing some type of justification for why a CLD could be interpreted as a network (e.g.,^8,23–25,27–30,37,42,43,47,50,54^).

It is important to note that even “off-the-shelf”^65^ network analysis metrics that are increasingly readily available in CLD building tools are subject to these conflicts. In addition, if network analysis is elected as the method to identify leverage points based on a CLD despite these conflicts, metrics should still not be used without reflection on what they are exactly measuring. For example, in the Python package NetworkX, the function to compute closeness centrality assumes that its users are looking for the ‘influenced’ or ‘collector of causal power’ rather than the ‘influencer’ or ‘spreader of causal power’, meaning that users attempting to generate results for the latter application are required to adapt the function themselves^76^. To facilitate such reflection, if network analysis metrics are used, as a minimum requirement, the equations used to compute the metrics should be given—which is currently not always the case.

From our evaluation, it however seems clear that we should refrain from using betweenness- and closeness centrality to identify leverage points based on CLDs. It may be possible to select or develop other network analysis metrics that are at least a better match with CLDs. Eigenvector centrality appears to have less restrictive assumptions^65^ and was shown to correlate with causal influence in directed acyclic graphs^59^. A step in the right direction could also be the addition of edge weights, which allow a modeller to indicate a larger or faster causal effect with higher weights^19,58,61,77^. Incorporating edge weights in CLDs could result in network analysis metrics being more consistent across CLDs that represent the same system and has the potential to mitigate problems with node exchangeability. Polarity could be accounted for as well with negative edge weights^58,77^. Methods to identify an optimal set of important nodes rather than a single important node have also been developed^78^.

Still, technical adjustments such as the incorporation of edge weights do not automatically make network analysis correspond better with the system dynamics perspective. The use of betweenness- and closeness centrality to identify leverage points based on CLDs, for example, relies on the assumption that causal power gradually diminishes while going down a causal chain^8^. This assumption warrants further scrutiny in its own right, especially because in the systems literature it is postulated that it is not just the *strength of association*, which assumes that causes further away from the outcome have increasingly weaker effects on system-level behaviour, but rather the *structure*, which assumes that even causes further away from the outcome can have significant effects on system-level behaviour due to feedback loops, that determines system-level behaviour^20^. Furthermore, network analysis can only interpret a CLD as describing causal effects e.g., of a factor X on a factor Y (pairwise interaction)—for example, less cars (X) lead to more cycling (Y). It cannot accommodate the case where the causal effect of X on Y depends on the value of a factor Z (higher-order interaction), i.e., a conditional causal effect that depends on interaction between X and Z—for example, less cars (X) lead to more cycling (Y) only if the built environment accommodates cycling (Z). That means that even if a CLD with edge weights that reflect ‘the amount of causal impact’ made by each of the included causal effects could be formulated, this in combination with network analysis would still be a limited representation of reality as it only allows for pairwise and not for higher-order interactions^79,80^—while computational modelling approaches can account for higher-order interactions by combining a set of factors in one equation.

Note that the actions required to transform a system and change system-level behaviour may go beyond what is suggested by the term ‘leverage *point*’, which seems to imply that we should identify and intervene on individual factors in CLDs. Still, in her description of high impact leverage points or “places to intervene in a system”, Meadows—originator of most of systems thinking’s core concepts—calls attention to “regulating negative feedback loops”, “driving positive feedback loops”, “the rules of the system”, “the goals of the system”, and “the mindset or paradigm out of which the system arises”^81^, where arguably none of these can be found in individual factors in the network of causes and effects that describes how a system operates. In this sense, the definition of the term ‘leverage point’ as it currently tends to be used in the literature relating to the development and analysis of CLDs may indeed be too narrow^82^.

As such, selecting or developing other network analysis metrics is not just a matter of technicalities, but also a matter of conceptual clarification, where the question is whether network analysis metrics pay sufficient regard to core characteristics of the system dynamics perspective, such as the importance of mental models, interactions between lower and higher domains, feedback loops, and conditional causal effects. Network analysis accordingly does not seem to be an adequate alternative to computational modelling approaches to quantitatively identify leverage points based on CLDs. On those grounds, efforts have been and should be made to facilitate the development of computational models on the basis of CLDs and to overcome some of the described challenges. For example, to enable computational modelling qualitative expert knowledge could be leveraged to ‘fill in the blanks’ left by the lack of quantitative data available on environmental factors^21^. Moreover, CLDs could be refined so that conditional causal effects are made explicit, the recording of which can support subsequent computational modelling steps because it facilitates the conversion to equations^21^.

## Conclusion

We conclude that using network analysis to identify leverage points based on CLDs leads to false inference. We have shown that the perception that network analysis, because of its ease of use, is an attractive alternative to computational modelling approaches to quantitatively identify leverage points based on CLDs is inaccurate, which is exemplified by the bodies of literature dedicated to the potential problems with network analysis in other fields. The current practice of using betweenness- and closeness centrality to identify leverage points based on CLDs, which is gaining momentum as an accepted practice to quantitatively identify leverage points based on CLDs, is at best premature and at worst incorrect. It could cause us to wrongly identify a factor as a leverage point, which is problematic as the results provided by this method can, in current practice, form the foundation for policy recommendations about how to address complex problems. Other quantitative or qualitative approaches that better correspond with the system dynamics perspective must be explored.

## Methods

### Baseline causal loop diagram

The baseline CLD is a simplified variant of a previously published CLD^62^. It was developed based on specialised knowledge (public health, health inequalities, dietary behaviour, sociology, and anthropology) through interviews and corroborated and supported by literature. It details two feedback loops between the norm—what BMI is regarded as normal—and weight-related behaviour: specifically, between the norm and food intake and between the norm and physical activity. BMI refers to body mass index, which is a measurement used at the population level to indicate whether a person has a healthy weight relative to their height. It is computed as a person’s weight in kilograms divided by the square of their height in meters, i.e., kg/m^2^. Essentially, the baseline CLD shows that the group-level norm affects individual-level weight-related behaviour and vice versa, where if a higher BMI is regarded as normal it may be more difficult for people to adopt healthy weight-related behaviour, while a person’s weight-related behaviour is also conducive to the norm. The polarities in the causal structure are chosen so that the baseline CLD accommodates the scenario where BMI is larger than individual ideal BMI, which represents the BMI a person regards as appropriate (this scenario may also be referred to as the context of validity for the baseline CLD^21^). In this scenario, a person thus believes they should lose weight.

### Betweenness centrality and closeness centrality

We computed betweenness- and closeness centrality, the undirected as well as directed variants, for the factors in the five CLDs (using the Python package NetworkX^83^). Betweenness- and closeness centrality are defined according to a node’s position on short paths between other nodes^58^.

#### Betweenness centrality

A node with high betweenness centrality lies on the highest number of shortest paths between pairs of other nodes in the network^58^. Under the assumptions of knowing and taking the shortest path, what flows through the network often passes a node with high betweenness centrality—e.g., betweenness centrality indicates how often packages pass a station in a package delivery system^65^. For social networks, this has been interpreted as a node being a gatekeeper^66^ in the network, whose role is to allow what flows through the network to pass from one part of the network to the other^84^.

In CLDs, the factor with the highest betweenness centrality thus lies on the highest number of shortest causal chains between pairs of other factors in the CLD. This is hypothesised to make a factor with high betweenness centrality an important mediator (compare gatekeeper in social network analysis) in the system^8,24,29^.

The betweenness centrality of a node $v$ is the sum of the fraction of the shortest paths between pairs of other nodes in the network that pass through $v$. This is computed as:

$${BC}_{v}=\frac{1}{(n-1)(n-2)}\sum_{s,t\in V}\frac{\sigma (s,t|v)}{\sigma (s,t)}$$

where $$V$$ is the set of nodes in the network, $$\sigma (s,t)$$ is the number of shortest paths between a node $$s$$ and a node $$t$$ and $$\sigma (s,t|v)$$ is the number of those paths passing through node $$v$$ (which is not equal to node $$s$$ or node $$t)$$. $$\frac{2}{(n-1)(n-2)}$$ is used to normalise for undirected networks (undirected variant), whereas $$\frac{1}{(n-1)(n-2)}$$ is used to normalise for directed networks (directed variant), where $$n$$ is the number of nodes in the network. This difference in normalisation comes from the premise that in an undirected network each path between a node $$s$$ and a node $$t$$ can be taken in two directions (from node $$s$$ to node $$t$$ and vice versa) and thus counts twice, whereas in a directed network each path between a node $$s$$ and a node $$t$$ can be taken only in one direction and thus counts once.

Note that the results for the undirected and directed variants of ${BC}_{v}$ differ because arrows can cause some paths between a node $s$ and a node $t$ to no longer be possible (i.e., if the path is now ‘blocked’ by an arrow pointing in the opposite direction).

#### Closeness centrality

A node with high closeness centrality on average has the shortest paths to other nodes in the network^58^. Under the assumptions of knowing and taking the shortest path, what flows through the network from a node with high closeness centrality quickly reaches other nodes in the network—e.g., closeness centrality indicates how long it takes for packages to arrive when they are sent from a station in a package delivery system^65^. For social networks, this has been interpreted as a node being an influencer^85^ in the network, from which what flows through the network quickly reaches the rest of the network^58^.

In CLDs, the factor with the highest closeness centrality on average has the shortest causal chains to other factors in the CLD. This is theorised to make a factor with high closeness centrality an important spreader of causal power (compare influencer in social network analysis) through the system^8,29^.

The closeness centrality of a node $v$ is the inverse of the average shortest path distance to $v$ over all $n-1$ nodes in the network reachable from $v$. This is computed as:

$${CC}_{v}=\frac{n-1}{{\sum }_{v=1}^{n-1}d(u,v)}$$

where $d(u,v)$ is the shortest path distance between a node $u$ and node $v$.

Note that the results for the undirected and directed variants of ${CC}_{v}$ differ because arrows can extend the shortest path distance $d(u,v)$ between a node $u$ and node $v$ due to some paths being no longer possible (i.e., if the path is now ‘blocked’ by an arrow pointing in the opposite direction).

No human participants were involved in the study.

### Supplementary Information

Supplementary Table S1.

## Data Availability

All data generated or analysed during this study are included in this published article.

## Abbreviations

BMI: Body mass index
CLD: Causal loop diagram
