# Supplementary material for: Using network analysis to identify leverage points based on causal loop diagrams leads to false inference
Source: Sci Rep. 2023 Nov 29;13:21046. doi: 10.1038/s41598-023-46531-z (PMC10687004; doi:10.1038/s41598-023-46531-z)
Supplement: Supplementary file 1 — Supplementary Table S1. [file 41598_2023_46531_MOESM1_ESM.pdf]

SUPPLEMENTARY INFORMATION

USING NETWORK ANALYSIS TO IDENTIFY LEVERAGE POINTS BASED ON CAUSAL LOOP DIAGRAMS LEADS TO FALSE INFERENCE

Loes Crielaard[1,2]*, Rick Quax[2,3], Alexia D. M. Sawyer[1,4], Vítor V. Vasconcelos[2,3,5,6], Mary Nicolaou[1,2,6], Karien Stronks[1,2,6], Peter M. A. Sloot[2,3,6]

[1] Amsterdam UMC location University of Amsterdam, Department of Public and Occupational Health, Amsterdam, The Netherlands [2] Institute for Advanced Study, University of Amsterdam, Amsterdam, The Netherlands [3] Computational Science Lab, Informatics Institute, University of Amsterdam, Amsterdam, The Netherlands [4] MRC Epidemiology Unit, University of Cambridge School of Clinical Medicine, Institute of Metabolic Science, Cambridge, United Kingdom [5] POLDER, Institute for Advanced Study, University of Amsterdam, Amsterdam, The Netherlands [6] Center for Urban Mental Health, University of Amsterdam, Amsterdam, The Netherlands

*Supplementary Table S1. The full factor rankings for the five CLDs on betweenness- and closeness centrality (the undirected and directed (indicated with *) variants).*

Betweenness centrality	baseline				(i) specify mediators				(ii) specify mediators and parameters				(iii) simplify				(iv) prune redundant factors			
Factor	Between-ness	Rank	Between-ness*	Rank	Between-ness	Rank	Between-ness*	Rank	Between-ness	Rank	Between-ness*	Rank	Between-ness	Rank	Between-ness*	Rank	Between-ness	Rank	Between-ness*	Rank
age	-	-	-	-	-	-	-	-	0	11	0	11	-	-	-	-	-	-	-	-
basal metabolic rate	-	-	-	-	0	8	0,022	7	0,154	6	0,071	9	-	-	-	-	-	-	-	-
BMI	0,196	4	0,339	3	0,367	1	0,456	1	0,378	1	0,333	3	0,143	4	0,310	4	0,119	3	0,333	2
discrepancy between BMI and individual ideal BMI	0,304	3	0,446	1	0,333	2	0,433	2	0,340	2	0,359	2	0,238	3	0,405	1	-	-	-	-
food intake	0	6	0,134	6	-	-	-	-	-	-	-	-	-	-	-	-	0,048	5	0,131	5
group-level BMI	0,143	5	0,179	5	0,156	5	0,156	5	0,141	7	0,128	8	0,143	4	0,190	6	0,095	4	0,190	4
healthy BMI	0	6	0	7	0	8	0	8	0	11	0	11	0	5	0	7	0	6	0	6
healthy weight-related behaviour	-	-	-	-	-	-	-	-	-	-	-	-	0	5	0,357	3	-	-	-	-
height	-	-	-	-	-	-	-	-	0	11	0	11	-	-	-	-	-	-	-	-
individual ideal BMI	0,393	1	0,357	2	0,311	3	0,311	3	0,244	4	0,205	4	0,429	1	0,381	2	0,500	1	0,381	1
norm	0,321	2	0,268	4	0,244	4	0,233	4	0,179	5	0,167	6	0,381	2	0,286	5	0,381	2	0,286	3
physical activity	0	6	0,134	6	-	-	-	-	-	-	-	-	-	-	-	-	0,048	5	0,131	5
physical activity level	-	-	-	-	0,033	7	0,067	6	0,077	8	0,051	10	-	-	-	-	-	-	-	-
socio-cultural ideal BMI	0	6	0	7	0	8	0	8	0	11	0	11	0	5	0	7	0	6	0	6
total daily energy expenditure	-	-	-	-	0,044	6	0,156	5	0,064	9	0,154	7	-	-	-	-	-	-	-	-
total daily energy intake	-	-	-	-	0	8	0,233	4	0,045	10	0,199	5	-	-	-	-	-	-	-	-
weight	-	-	-	-	-	-	-	-	0,288	3	0,385	1	-	-	-	-	-	-	-	-

Closeness centrality	baseline				(i) specify mediators				(ii) specify mediators and parameters				(iii) simplify				(iv) prune redundant factors			
Factor	Close-ness	Rank	Close-ness*	Rank	Close-ness	Rank	Close-ness*	Rank	Close-ness	Rank	Close-ness*	Rank	Close-ness	Rank	Close-ness*	Rank	Close-ness	Rank	Close-ness*	Rank
age	-	-	-	-	-	-	-	-	0,271	11	0,171	11	-	-	-	-	-	-	-	-
basal metabolic rate	-	-	-	-	0,417	7	0,246	6	0,361	7	0,178	9	-	-	-	-	-	-	-	-
BMI	0,571	2	0,409	1	0,588	1	0,457	1	0,520	1	0,297	1	0,538	3	0,397	1	0,538	3	0,446	1
discrepancy between BMI and individual ideal BMI	0,615	1	0,281	3	0,588	1	0,305	3	0,500	2	0,231	3	0,583	2	0,238	4	-	-	-	-
food intake	0,444	4	0,300	2	-	-	-	-	-	-	-	-	-	-	-	-	0,538	3	0,298	4
group-level BMI	0,533	3	0,237	8	0,500	3	0,213	10	0,419	4	0,173	10	0,538	3	0,238	4	0,538	3	0,275	6
healthy BMI	0,400	5	0,255	5	0,357	8	0,245	7	0,302	9	0,192	6	0,412	5	0,245	3	0,438	4	0,303	3
healthy weight-related behaviour	-	-	-	-	-	-	-	-	-	-	-	-	0,438	4	0,298	2	-	-	-	-
height	-	-	-	-	-	-	-	-	0,351	8	0,248	2	-	-	-	-	-	-	-	-
individual ideal BMI	0,615	1	0,265	4	0,526	2	0,267	4	0,419	4	0,208	5	0,636	1	0,238	4	0,700	1	0,325	2
norm	0,533	3	0,250	6	0,476	4	0,237	8	0,382	6	0,189	7	0,583	2	0,238	4	0,636	2	0,298	4
physical activity	0,444	4	0,300	2	-	-	-	-	-	-	-	-	-	-	-	-	0,538	3	0,298	4
physical activity level	-	-			0,435	6	0,256	5	0,419	4	0,189	7	-	-	-	-	-	-	-	-
socio-cultural ideal BMI	0,364	6	0,245	7	0,333	9	0,225	9	0,283	10	0,179	8	0,389	6	0,245	3	0,412	5	0,286	5
total daily energy expenditure	-	-	-	-	0,435	6	0,320	2	0,394	5	0,223	4	-	-	-	-	-	-	-	-
total daily energy intake	-	-	-	-	0,455	5	0,320	2	0,419	4	0,231	3	-	-	-	-	-	-	-	-
weight	-	-	-	-	-	-	-		0,464	3	0,297	1	-	-	-	-	-	-	-	-
